# The Use of a Modular Titanium Baseplate with a Press-Fit Keel Implanted with a Surface Cementing Technique for Primary Total Knee Arthroplasty

**DOI:** 10.1155/2014/972615

**Published:** 2014-05-14

**Authors:** Christopher E. Pelt, Jill Erickson, Bryt A. Christensen, Benjamin Widmer, Erik P. Severson, David Evans, Christopher L. Peters

**Affiliations:** Department of Orthopaedics, University of Utah, 590 Wakara Way, Salt Lake City, UT 84108, USA

## Abstract

Little data exists regarding outcomes following TKA performed with surface-cementation for the fixation of modular tibial baseplates with press-fit keels. Thus, we retrospectively reviewed the clinical and radiographic outcomes of 439 consecutive primary TKAs performed with surface cemented tibial components. There were 290 female patients and 149 male patients with average age of 62 years (range 30–84). Two tibial components were revised for aseptic loosening (0.5%) and four tibial components (0.9%) were removed to improve instability (*n* = 2) or malalignment (*n* = 2). Complications included 13 deep infections treated with 2-stage revision (12) and fusion (1). These results support the surface cement technique with a modular grit-blasted titanium surface and cruciform stem during primary TKA.

## 1. Introduction

Although results of tibial component fixation with cement in total knee arthroplasty (TKA) have been good, [1–14] the ideal technique of cementing is still debated [15–18]. Full cementation of the tibial component (cementation of the undersurface of the tibial component and stem) has been advocated to improve short-term and long-term fixation of the tibial component [2, 18–20].

Surface cementation (solely cementing the undersurface of the tibial component and press fitting of the tibial stem) has been reported to afford sufficient implant stability with the potential for decreased metaphyseal bone loss on revision and decreased stress shielding associated with the cemented stem [15, 17, 21–24]. Previous biomechanical studies have demonstrated that surface cementation does not increase early micromotion compared to fully cemented baseplates, assuming adequate cement penetration in the proximal tibial surface [21, 25].

The purpose of this investigation was to demonstrate the clinical outcomes including Knee Society scores and complications/revisions as well as radiological outcomes of a modular tibial base plate implanted with a surface cemented technique.

## 2. Materials and Methods

After institutional review board approval, we retrospectively reviewed the surgical database of a single experienced surgeon at a major academic institution. We identified 962 consecutive primary TKAs performed between 2000 and 2007 that underwent primary TKA. Patients were included in the study, if they had received a modular titanium baseplate with a cruciform keel using a surface cemented technique and had a minimum five-year followup or failure of the TKA prior to five years. Failure was defined as revision for any reason; all patients that failed regardless of the length of followup remained in the analysis. Sixty patients (65 knees) died prior to a five-year followup and had not undergone revision TKA at the last followup; these patients were removed from the analysis. Additionally, 458 knees did not meet the inclusion criteria and had not failed at last followup and were therefore excluded. This resulted in a final series of 439 knees from 351 patients.

**Figure 1:**
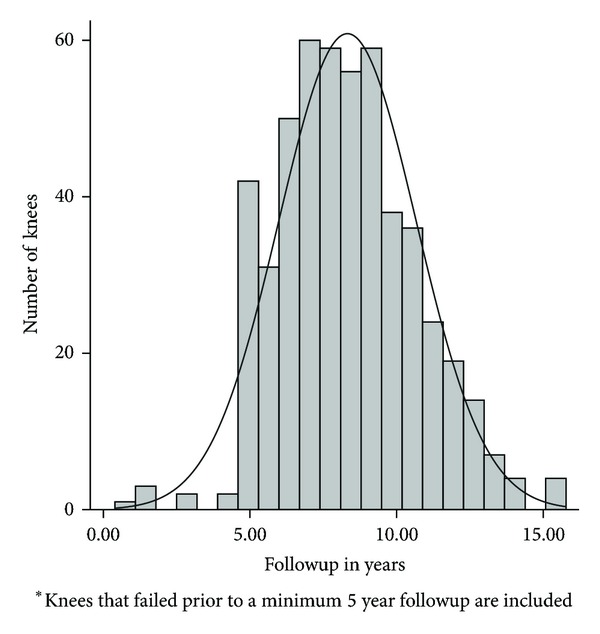
Histogram demonstrating the number of patients available for followup.

Patients were followed according to a prospective clinical and radiographic protocol for primary and revision TKA that has been consistently used at our institution since 1995, with scheduled followup at 2 weeks, 6 weeks, 6 months, 1 year, and every 1-2 years thereafter. Mean followup was 8 years, including those who failed prior to five years (range, 0.4–15.8, Figure 1). The clinical scores and implant records were obtained from the database. Clinical results were graded according to the Knee Society Clinical Rating System by a member of the research team other than the operating surgeon [26]. For this study, the preoperative and most recent follow-up knee scores were compared, as well as documentation of the number of failures, reoperations, and complications. Long-standing anteroposterior view of lower extremities and weight-bearing anteroposterior, lateral and merchant radiographs were obtained at six weeks. The same films were taken at each followup without the long-standing view. Radiographs were evaluated by one of the authors according to the Knee Society Total Knee Arthroplasty Roentgenographic Evaluation and Scoring System [27] for radiolucent zones, femoral component flexion and valgus, tibial component slope and varus, and anatomic axis was measured on the longstanding film for overall mechanical alignment.

### 2.1. Surgical Methods

Surgery was either performed under general or spinal anesthetic with a femoral nerve block (after 2004). A standard medial parapatellar approach was utilized for all cases. The femoral preparation utilized intramedullary referencing with a goal of four to five degrees of valgus relative to the anatomic axis of the femur for the distal cut. The remainder of the femoral preparation was performed with the use of a standard posterior referencing guide with the goal of rotational alignment being parallel to the epicondylar axis using a measured resection technique. Similar condylar design femoral components were used in all cases (Maxim or Vanguard; Biomet, Warsaw, IN).

**Figure 2:**
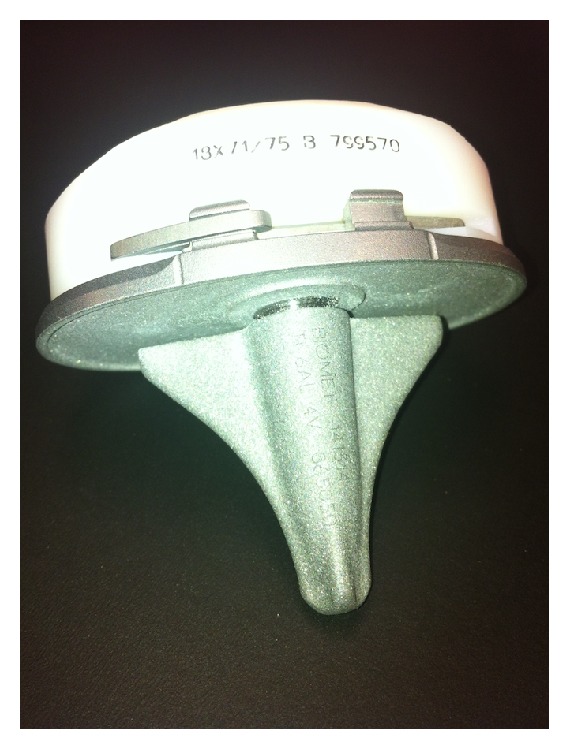
A modular titanium Maxim (Biomet, Warsaw, IN, USA) tibial baseplate.

**Figure 3:**
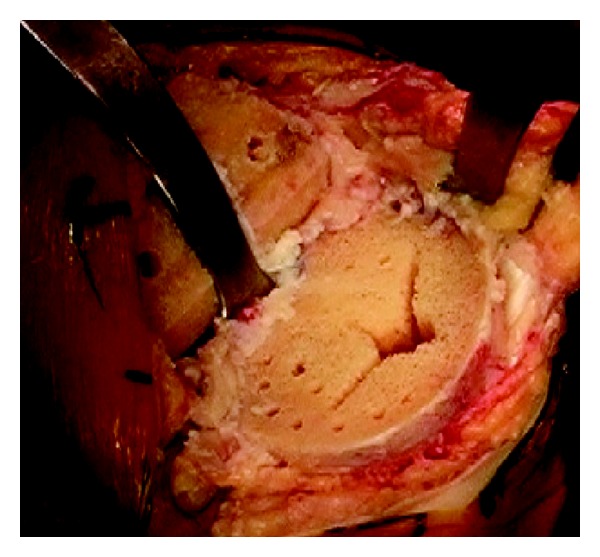
Tibial surface after preparation with drilling sclerotic bone and pulse lavage.

**Figure 4:**
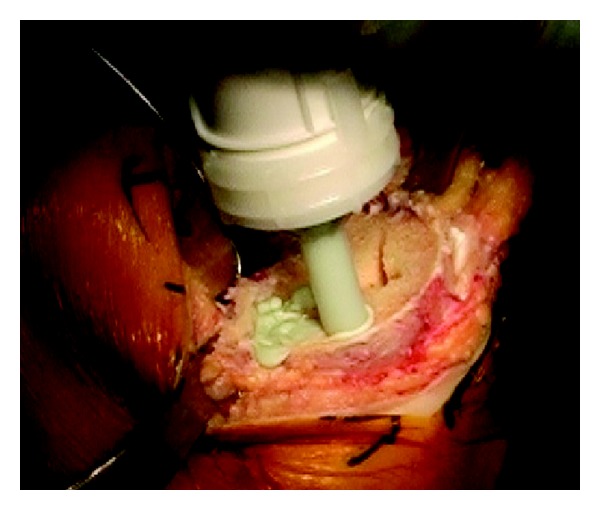
Pressurization of cement with nozzle into cut tibial surface.

The tibial preparation was performed with an extramedullary cutting guide referencing 6–8 mm off the less affected side of the tibial plateau with an alignment goal perpendicular to the mechanical axis of the tibia. After the cut surface of the tibia was checked for proper alignment, the tibia was broached with a cruciform tibial punch slightly undersized from the real tibial keel to achieve true press fit. The tibial implant consisted of a modular titanium baseplate and cruciform stem with a grit-blasted titanium surface with an average surface roughness of 6.8 *μ*m (Maxim; Biomet, Warsaw, IN) (Figure 2). In order to ensure adequate depth of cement penetration, a 2.0 mm drill was used to drill sclerotic areas of the tibia to a depth of 5 mm (Figure 3). Pulsatile lavage was then used for final surface preparation. Cefuroxime antibiotic powder (750 mg) was mixed dry with Palacos (multiple vendors) or cobalt cement (Biomet, Warsaw, IN) powder by the surgical technician. When appropriate, the mixture was combined with the fluid component, mixing for one minute by hand, and then allowed to sit for one minute, after which it was applied to the undersurface of the tibial baseplate in a low-viscosity state. The remaining cement was then pressurized into the cut surface of the tibia using a cement gun with a short nozzle with a goal for cement penetration of 3–5 mm (Figure 4). The tibial components were paired with a fixed cruciate-retaining polyethylene bearing in 78% of cases, a fixed posterior-stabilized bearing in 15%, and a fixed anterior-stabilized bearing in 7%.

### 2.2. Statistical Methods

Clinical improvement in the Knee Society Score was analyzed using the Wilcoxon Signed Rank Test. Descriptive statistics are reported as means and ranges or 95% confidence intervals for continuous data and proportions for binary data. Data was analyzed using STATA v13.1 (College Station, Texas, USA). Values less than *P* = 0.05 were considered statistically significant.

## 3. Results

Of the 439 knees, 290 were female and 149 were male. The average age was 62 years (range, 30–84 years) and the average body mass index (BMI) was 33.07 kg/m^2^ (range, 18.6–64.2 kg/m^2^). Mortality in this patient population was <1% (*n* = 2).

**Figure 5:**
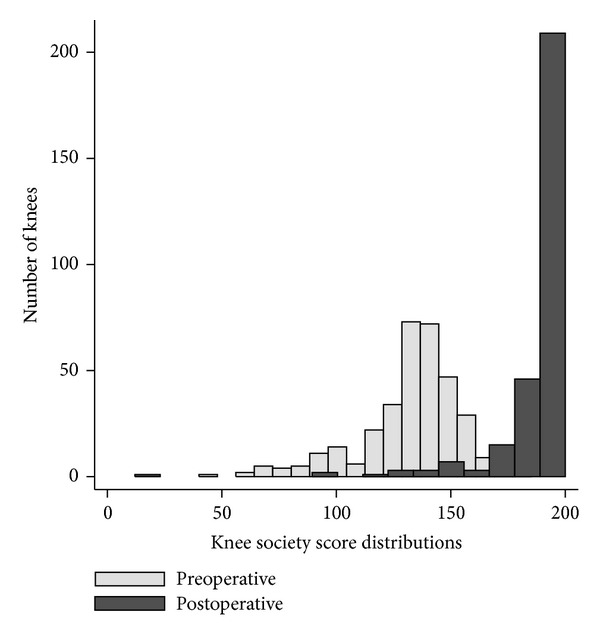
Histogram demonstrating the preoperative versus last followup scores for the Knee Society score.

Knee Society scores at last followup were compared to those obtained preoperatively (Figure 5) and demonstrated a lasting statistically significant improvement in total Knee Society scores (preoperative mean total score 132 (95% CI 130–134), postoperative mean total score 189 (95% CI 186–191), *P* < 0.001).

Nineteen surface cemented tibial components were removed (4%), six for aseptic revision and thirteen for sepsis. Two knees (0.5%) were revised for suspected aseptic tibial component loosening. Of these, one was revised at 4.84 years postoperatively and the other at 2.45 years postoperatively. Four (0.9%) knees had well-fixed tibial components removed to correct instability, which required increased constraint, or to correct maltracking/malalignment. Thirteen (3%) tibial components were removed for treatment of deep infection requiring two-stage revision in twelve knees and fusion in one knee. The tibial component was retained in 420 of 439 knees (96%).

**Figure 6:**
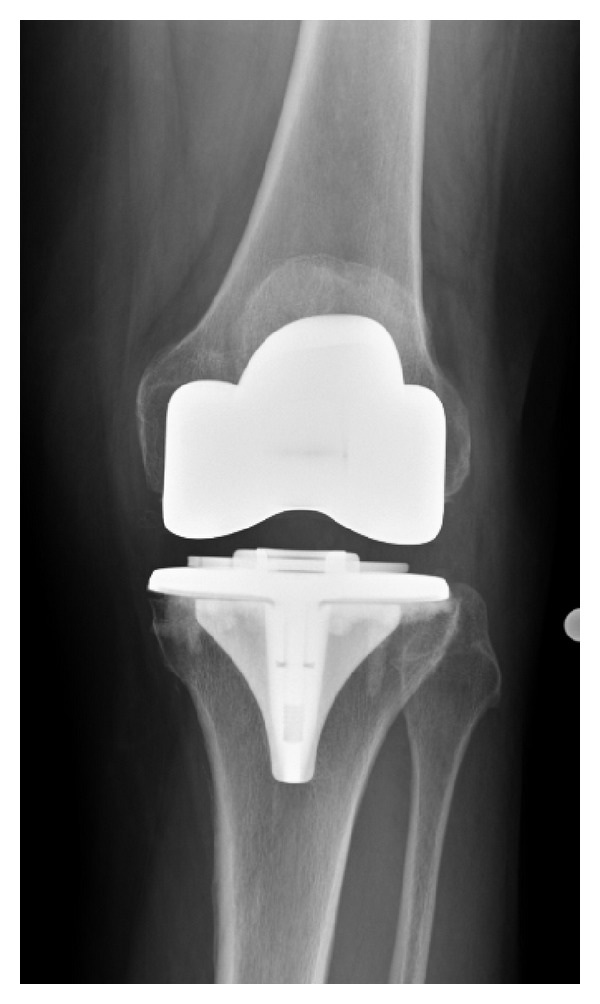
Standing anteroposterior projection of a surface cemented arthroplasty.

**Figure 7:**
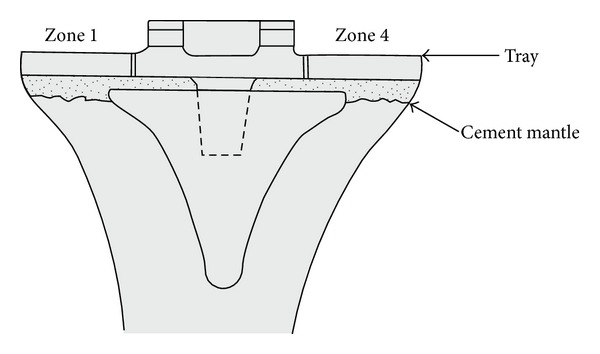
The location and number of radiolucencies visualized under the tibial component using the Knee Society Total Knee Arthroplasty Roentgenographic Evaluation and Scoring System.

A radiographic review of all 439 knees revealed a mean femoral component valgus of 5 degrees, femoral component flexion of 5 degrees, tibial varus of 2 degrees, and tibial slope of 2 degrees. The average anatomic axis, measured on longstanding postoperative radiographs, was 4 degrees of valgus (Table 1). Postoperative radiographs demonstrate the radiographic appearance of surface cementing technique in Figure 6. Radiolucent zones were found in Zone 1 in three knees (<1%) and in Zone 4 in 7 knees (1.5%) (Figure 7). In one knee with two zones with radiolucency, the component did not show evidence of loosening or subsidence and the patient had no complaints of knee pain. No knees had lucencies on a lateral radiograph or complete radiolucencies, and thus no tibial component was deemed radiographically loose. Retention of the tibial component during revision of the femur, patella, or polyethylene insert occurred in 18 (4.1%) knees during the follow-up period.

## 4. Discussion

Although support for the technique of surface cementation with press-fit designs of the tibial component exists in the literature, [24, 28] other studies have demonstrated higher failure rates with this technique [20, 29]. Sharkey et al. [20] report an association between early loosening and uncemented as well as surface cemented implants and recommended that the surface cementing technique should be abandoned. However, they report that overall, only 10.5% of patients that failed with aseptic loosening were surface cemented; the others were either uncemented (21%) or fully cemented (68.5%). Additionally, Arora and Ogden [29] felt that the surface cementing technique may have been a contributor, along with polyethylene wear and a rotationally loose patella, to the high rate of osteolysis seen in their study. Given a lack of consensus in the literature, we set out to determine our experience using a modular titanium tibial baseplate implanted with surface cementation technique. This longitudinal series of a large number of TKAs demonstrates that the surface cement technique in the setting of total knee arthroplasty can be performed safely and effectively. Two cases of suspected aseptic loosening of the tibial component were identified and very few knees demonstrated radiographic radiolucencies. Faris et al., [30] using a similar component and surface cement technique, demonstrated 97.2% survival of TKAs out to 13 years. Two of five revisions performed (of 201 primary TKAs) were a result of aseptic tibial component failures resulting from medial tibial collapse (1%). Hofmann et al. [24] reported 98% survival of TKAs performed with a similar technique with minimum 5-year followup, with no revisions due to a loose tibial component.

There are limitations of the current study. First, the retrospective study design imparts the possibility for selection bias. There was a high loss of followup, in the part related to the large geographic region of referral for our tertiary academic medical center. We attempted to contact all patients by telephone and electronic medical records review. We included all known failures that occurred in less than 5 years, but patients otherwise lost prior to completion of five-year followup were excluded, which would likely account for a greater proportion of failures in our cohort, given that the majority of revisions return to our center. In addition, there may be some selection bias against the use of the surface cemented technique in knees with bone loss or poor bone quality by the surgeon, in which cases a fully cemented tibial component, instead of surface cemented, may have been used. We identified only 3.4% of TKAs that received fully cemented components during the study period, suggesting that the impact of this selection bias is likely relatively minor but important to note, given that the results of our study may also not be generalizable to patients with significant proximal tibial bone loss, cysts, or severe bone density concerns. Finally, our conclusions must be limited to similar technique and implants. It is possible that a similar technique with other implant designs (nonmodular, smooth, cobalt-chrome or all polyethylene tibias, etc.) may not show similar results, and we caution against the generalization of our findings to all implants.

The conflicting surgical results reported with surface cementation in the literature suggest that several variables including implant design, surgical technique, and tibial bone remodeling or response may play a role in determining implant survival. Implant design has been shown to be a critical factor influencing the success of surfaced cemented tibial components. For example, one of the most widely cited studies critical of surface cementation exhibited high failure rates with a macrotextured (waffle-pattern) tibial base plate [31]. The idea that this particular implant design may be ill-suited for surface cementation was illustrated in a biomechanical study by Pittman et al. [32], wherein it was shown that the interface strength increased against both tensile and torsional stress as the tibial baseplate surface roughness became coarser, except in the case of a “waffle-pattern,” or macrosurfaced baseplate which proved to be vulnerable to failure with rotational loading. This same study demonstrated a stronger but not significant difference in the bond between titanium and cobalt-chromium alloys. Bundy and Penn [33] also report on the surface preparation and suggest that both roughened surfaces and highly polished surfaces improve the metal and bone cement interface. They suggest that altering the surface topography and roughness is the most effective way to change the strength of the metal and bone interface.

Surgical technique, including tibial surface preparation as well as cement pressurization or penetration into the cut surface, is likely also important. Prior biomechanical studies have supported that the surface cement technique affords similar baseplate fixation as compared to the fully cemented technique, including eccentric loading as may be seen in varus and valgus alignment [15, 24, 25, 32, 34]. Peters et al. [25] showed no difference in the micromotion between the surface cemented and the fully cemented techniques, but there was a significant correlation between the initial fixation stability of the tibial implant and the depth of cement penetration into the cancellous bone of the proximal tibia. Similarly Bert and McShane [15] showed that if the cement mantle beneath the tibial base plate was 3 mm, excellent stability of the implant was seen, regardless of whether the stem was cemented. Several studies have also stressed the importance of optimal cement penetration to provide sufficient initial mechanical bond strength of the tibial base plate [15, 24, 25, 32]. More recently, the technique of finger packing cement in the tibia followed by impacting a precoated tibial component has been shown to lead to 3–5 mm of cement penetration, and cement gun pressurization, the method used in the present study, achieves approximately 5 mm of penetration [34]. In order to further enhance surface cement penetration, Dorr et al. [35] recommended pulsatile lavage and application of cement in a low-viscosity state, also techniques employed in this study. An additional important factor regarding tibial component fixation and technique is the response of host bone to the implant. Separate midterm in vivo studies support the concept that cementation of the stem or keel portion of the tibial component is associated with reduced density of bone in the tibial metaphysis, which may be associated with inferior component survival [36, 37]. Given these biomechanical studies and our clinical results, the authors utilize the surface cement technique commonly and make no alterations to postoperative rehabilitation protocols, allowing for immediate weight bearing as tolerated and rapid recovery techniques without compromise.

We have confirmed previous reports of good results using a surface cementation technique of the tibial component. If a properly designed implant is used in conjunction with proper surgical technique, early implant stability can be achieved. As the number of TKAs performed in North America increases exponentially in the next several decades, [38] continued effort to define optimum implant design and surgical technique will become increasingly important. Given the positive clinical and radiological outcomes seen in this series and only a small amount of complications demonstrated along with the theoretical advantages of improved proximal tibial bone density and less tibial bone loss at the time of revision, we feel that surface cementing the tibial component is a reasonable technique for consideration in total knee arthroplasty.

## Figures and Tables

**Table 1:** Radiographic measurements including femoral and tibial coronal and sagittal alignment, as well as overall anatomic valgus angle (hip knee ankle angle).

	Average	Minimum	Maximum	Standard deviation
Femur valgus	5°	−3°	16°	2°
Femur flexion	5°	−10°	14°	3°
Tibia varus	2°	−3°	7°	2°
Tibia slope	2°	3°	10°	2°
Anatomic axis (valgus)	4°	−7°	16°	3°
